# Construction of a novel digital method for quantitative analysis of occlusal contact and force

**DOI:** 10.1186/s12903-023-02899-y

**Published:** 2023-04-01

**Authors:** Zhe Zhao, Qing Wang, Jiale Li, Ming Zhou, Kai Tang, Jihua Chen, Fu Wang

**Affiliations:** State Key Laboratory of Military Stomatology & National Clinical Research Center for Oral Diseases, Department of Prosthodontics, School of Stomatology, The Fourth Military Medical University, Xi’an, Shaanxi 710032 PR China

**Keywords:** Digital, 3D, Occlusal analysis, Force analysis

## Abstract

**Background:**

Occlusal analysis is essential in the dental clinical practice. However, the traditional occlusal analysis performed on the two-dimensional level can not directly correspond to the tooth surface with three-dimensional profile, therefore the clinical guidance value is limited.

**Methods:**

By combining the 3D digital dental models and quantitative data from 2D occlusal contact analysis, this study constructed a novel digital occlusal analysis method. The validity and reliability of DP and SA were verified by comparing the results of occlusal analysis of 22 participants. ICC values for occlusal contact area (OCA) and occlusal contact number (OCN) were tested.

**Results:**

Results confirmed the reliability of the two occlusal analysis methods with ICC values of 0.909 for SA_OCA_, 0.906 for DP_OCA_, 0.929 for SA_OCN_ and 0.904 for DP_OCN_. The Bland-Altman plot, paired t-test (t_OCN_ = 0.691, *P* > 0.05) and Pearson correlation analysis results (*R* = 0.68, *p* < 0.001) verified the validity between SA and DP. Then a novel digital occlusal analysis method was constructed, which not only can locate the occlusion contact and provide the quantitative analysis, but also provide a comprehensive description of the resultant force of each tooth and the component forces on the *x*-, *y*- and *z*-axis.

**Conclusions:**

This new occlusal analysis method can obtain quantitative analysis of occlusal contact including contact area and force information simultaneously, which will provide new impetus and greater help for clinical dental treatment and scientific research.

## Background

Occlusion is closely related to oral health and considered as an important indicator of the functional status of the masticatory system [1, 2]. Occlusal analysis plays an important role in the diagnosis, treatment and prognosis evaluation in the field of prosthodontics [3, 4], orthodontics [5–7], implants [8], maxillofacial surgery [9], etc.

Every improvement in occlusal analysis methods has brought great changes to dental practice. Generally, the content of occlusion analysis should include occlusal contact area (OCA), occlusal contact number (OCN) and occlusal force. The articulating paper has always been a traditional tool for occlusal analysis, and is widely used in clinical practice because of its simplicity and intuitiveness. However, the size and distribution of occlusal contact can only be determined according to the doctor's experience [10]. It can not be used for quantitative analysis.

In recent years, the advent of occlusal analysis systems such as the T-scan system [11], Dental Prescale system [12], Blue Silicone system [13], etc. makes quantitative measurement possible. These systems use a media, such as silicone, PTE film or piezoelectric transducers, to analyze occlusal contact. For example, Dental Prescale occlusion analysis system uses a pressure-sensitive film with polyethylene terephthalate (PET) shells, which contains a pigment layer which consists of small dye capsules and a colour development reagent layer. When pressure is applied to film, the dye capsule ruptures and releases the colourless dye to mix with the substance in the colour development layer, thus presenting colour changes at where under the pressure [14]. The colour changes can be interpreted in the software to calculate the occlusal force value of each occlusal contact point. It can obtain the OCA values and absolute values of occlusal forces [15–17]. However, the data obtained on the two-dimensional level of these systems can not directly correspond to the tooth surface with three-dimensional profile, therefore the clinical guidance value is limited.

The rise of digital technology has brought new ideas to the development of dental analysis instruments [18–22]. The intra-oral scanners allow dentists to obtain three-dimensions digital models of the dentition directly [23, 24], with the advantages of high accuracy, enhanced patients’ comfort and possibility of quantitative analysis [25, 26]. As an important part of digital workflow, digital analysis software provides powerful function for the measurement of distances, areas, coordinates, errors, etc. [27, 28]. In recent years, the integration of digital technology into occlusal analysis has led to the development of occlusal analysis methods based on 3D dental models [29]. The advent of these methods has made it easier to quantify OCA, etc., however, the analysis of bite force is still lacking.

A combination of the 3D dental models obtained by intraoral scanning and quantitative data from 2D occlusal contact analysis, make it possible for quantitative analysis of occlusal contact and force simultaneously. This study constructed a novel digital occlusal analysis method which can not only be used to locate the occlusal contact on the tooth surface, but also to make quantitative analysis of OCA, OCN and occlusal contact force of each teeth, or part of dentition.

This study consists of two parts. Firstly, the consistency of used 2D and 3D methods was evaluated in vivo. After confirming the consistency, these two methods were used to construct a novel occlusal analysis system.

## Materials and methods

### Evaluation of the consistency of used 2D and 3D methods

#### Subjects

The evaluation trial enrolled twenty-two subjects (mean age 23.1 ± 3.2 years), all of whom were dental students at the first author's institution. All participants had complete dentition; no temporomandibular joint disease; no periodontal disease; and no orthodontic treatment. Ethical approval was granted by the Ethical Committee of the first author’s university (approval number: IRB-REV-2021143). All subjects gave informed consent before participating in the study.

#### Dental prescale analysis system (DP)

**Fig. 1:**
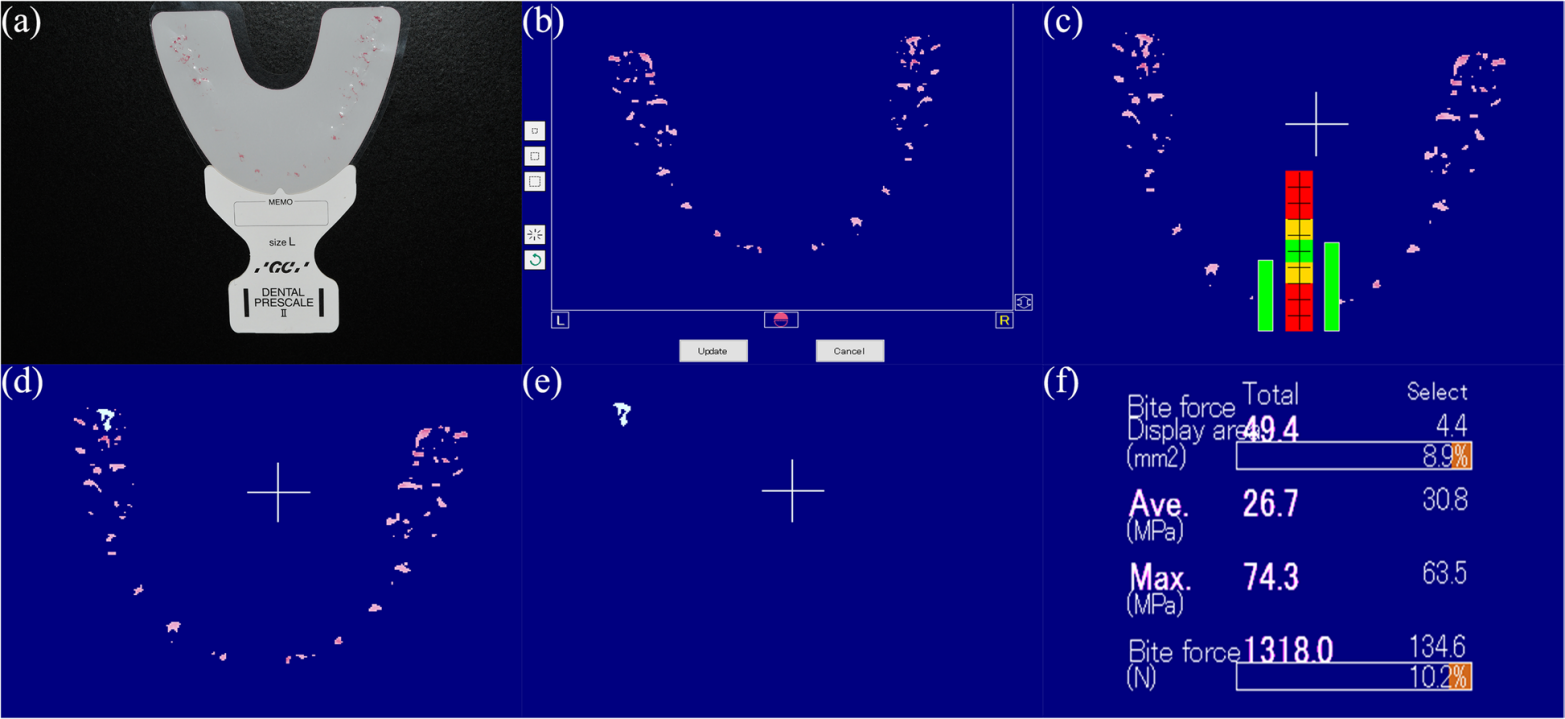
Occlusion analysis using the DP system. **a**. The pressure-sensitive film used in the DP system. **b**. Remove stray points that are significantly off the dental arch. **c**. 2D occlusal force analysis graph obtained from DP. **d-e.** Select the occlusal contact point. **f**. Measure the occlusal contact area and occlusal contact force

Before measurement, subject was asked to practice biting to find their intercuspal position (ICP). The pressure-sensitive films (Dental Prescale II, GC, Japan) are available in three sizes, S/ M/ L, and the appropriate size was selected according to the size of the subject's dental arch. The film was placed on the subject's mandibular arch, ensuring that it covered the entire dentition. The subject was then asked to bite with the maximum force into the ICP position and held for more than 3 s. After removing the saliva and drying, the film was immediately transferred to the scanning instrument (Perfection V600 Photo Scanner, Epson, Nagano, Japan) and the data were analyzed with the specific software (GC Bite Force Analyzer software, GC Corp.) to obtain a 2D occlusal contact graph, from which the OCA, OCN and occlusal force can be obtained (Fig. 1).

#### Scanning of articulating paper marks (SA)

**Fig. 2:**
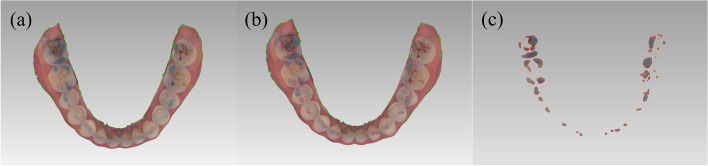
Occlusion analysis using the SA system. **a**. Digital model obtained by scanning. **b**. Outline the occlusal contact points. **c**. 3D occlusal analysis graph obtained from SA

Arch-shaped articulating paper (BK52, Bausch Germany GmbH, Hainspitz, Thüringen, Germany) was placed on the occlusal surface of the mandibular dentition. After ensuring that the articulating paper covered the entire dentition, the subject was instructed to bite with the maximum force into the ICP. After removing the articulating paper, the maxillary and mandibular dentition were scanned immediately using an intraoral scanner (Trios 3 Shape; 3Shape A/S, Copenhagen, Denmark) to obtain a 3D model with the colored marks of the articulating paper. The data were saved in "ply." Format and imported into a 3D data processing software (Geomagic Wrap 2017; 3D Systems, Rock Hill, SC, USA) where the teeth with occlusal marks could be observed. Each occlusal contact was outlined according to the color difference. The OCA and OCN values were obtained using the software's area measurement function (Fig. 2).

#### Reliability testing

For each method, a test–retest check was performed for all subjects two weeks after the initial test. All operations were performed by the same operator. Intraclass correlation coefficient (ICC) was used to evaluate the test–retest reliability for both systems with high ICC values representing high reliability.

#### Validity testing

Validity between the two systems was evaluated by comparing the OCA and the OCN obtained from DP and SA. The Shapiro–Wilk test was used to confirm the normality of the data sets. And then Paired t-tests were used to evaluate whether there was a significant difference in OCA and OCN obtained from DP and SA. Pearson correlation analysis was used to analyze the correlation between the OCA obtained from DP and SA. In addition, linear regression was used to evaluate the linear relationship between the data obtained from DP and SA.

Statistical analysis was performed using the SPSS v23 (SPSS Inc., Chicago, IL, USA), and GraphPad v8.0.2 (GraphPad Prism Software Inc., San Diego, CA, USA). For all analyses, statistical significance was preset at α = 0.05.

### Construction of the 3D occlusal force analysis system

#### Overlapping DP and SA records

**Fig. 3:**
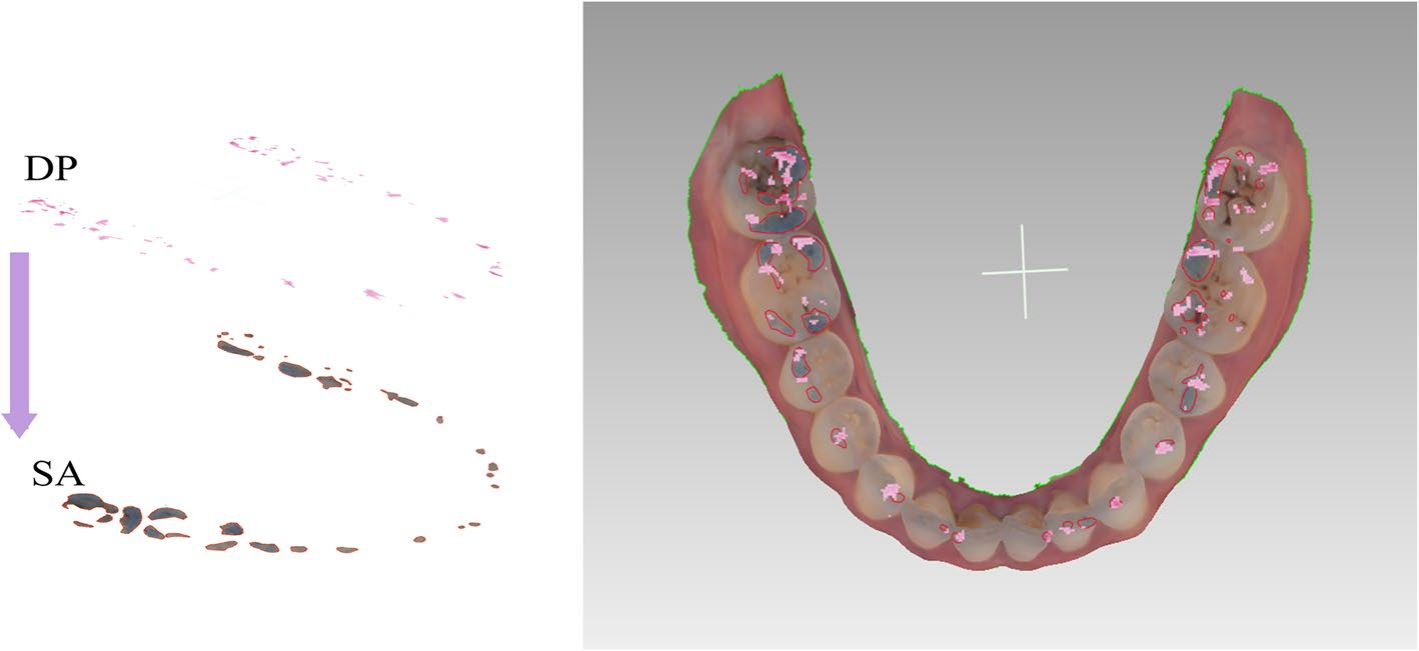
Overlapping DP and SA records

First, the 3D dental model with occlusal markers was rotated in Geomagic Wrap and its mandibular occlusal plane was adjusted to be parallel to the screen. Then, a screenshot was taken to obtain a 2D view of the occlusal plane of the 3D dental model. The 2D occlusal force analysis graph from Dental Prescale system and the screenshot of the occlusal surface of the 3D dental model were overlaid and the occlusal contact points measured by the two analysis systems were matched. The area of each occlusal contact point and the values of the occlusal forces were recorded (Fig. 3).

#### Construct a 3D occlusal force analysis model

**Fig. 4:**
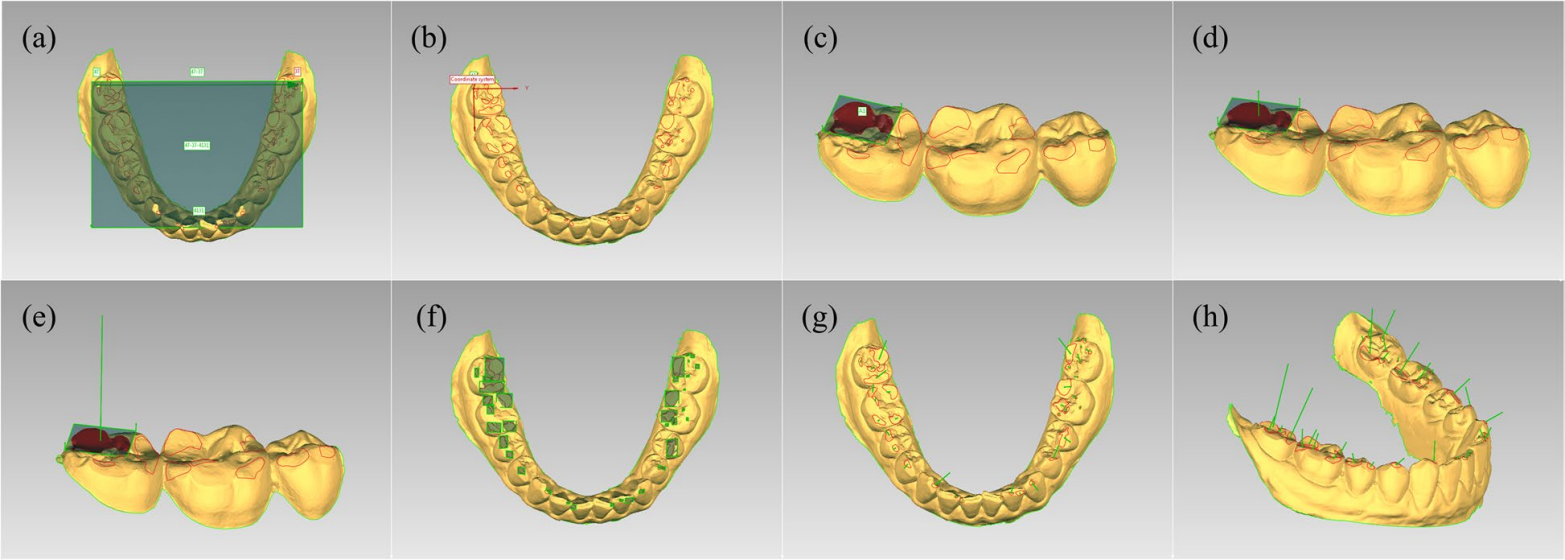
The process of constructing a 3D occlusal force analysis model. **a-b**. Construct the coordinate system. **c**. Construct a fitted plane. **d**. Set the occlusal force direction. **e**. Construct a occlusal force indicator segment. **f**. Fitting planes to the occlusal points of the entire dentition. **g**-**h**. 3D occlusal force analysis model of the entire dentition

Then, a 3D coordinate system was constructed in Geomagic Wrap software. The midpoint between the mesial line angles of the mandibular central incisors was taken as the origin. The *x*–*y* plane coincided with the mandibular occlusal plane, which contained the distobuccal cusps of the last molars and the origin. The x-axis passed through the origin and was perpendicular to the line of the distobuccal cusps of the last molars, with the positive *x*-axis pointing anteriorly. The *y*-axis passed through the origin and was parallel to the line of the distobuccal cusps of the last molars, with the positive external axis pointing to the left. The *z*-axis passed through the origin and is perpendicular to the *x*–*y* plane, with the positive *z*-axis pointing upwards (Fig. 4a-b).

Following, we presented an example of occlusal force model construction using the occlusal contact point (A_1_) on the mandibular second molar. First, the plane fitting function was used to obtain the fitted plane for each occlusal contact point (Fig. 4c). The opposite direction of the normal of the fitted plane was the direction of the occlusal force on this occlusal contact point (Fig. 4d). Using the center of the fitting plane as the base point and the absolute value of the bite force as the length, a line segment was constructed along the direction of the bite force (Fig. 4e). The coordinate values of the two end points of this line segment were noted as (x_1_, y_1_, z_1_) and (x_2_, y_2,_ z_2_). The diff. of the *x*-coordinates of the two endpoints (x_1_-x_2_) was the magnitude of the component force in the *x*-axis direction of the bite force on this occlusal contact point. Similarly, the diff. of the *y*-coordinate (y_1_-y_2_) and the *z*-coordinate (z_1_-z_2_) of the two endpoints was the magnitude of the component forces of the occlusal force on the *y* and *z* axes.

By adding up the values of the occlusal component forces in the *x*-axis direction for all occlusal contact points on the second molar, the value of the component forces in the *x*-axis direction to which the second molar was subjected can be obtained. The component forces on the second molar in the *y*-axis and *z*-axis can also be calculated in the same way. The forces on the other teeth can be analysed in the same way to create a 3D occlusal force analysis model of the entire mandibular dentition (Fig. 4f-h).

#### Clinical application of the 3D occlusal force analysis system

The clinical demonstration was carried out on a patient (30 years old) with bruxism. She had complete dentition; no periodontal disease; and no orthodontic treatment. Ethical approval was granted by the Ethical Committee of the first author’s university (approval number: IRB-REV-2021143). She gave informed consent before participating in the study.

## Results

### Evaluation of the reliability of SA and DP

The OCA, OCN and the corresponding ICC values of two methods are summarized in Table 1. The ICC values for OCA and OCN measured by both the SA and DP systems were higher than 0.9, so it can be confirmed that both analysis systems have a high reliability.

**Table 1:** Mean OCA values, OCN values and the intraclass correlation coefficient (ICC) of SA and DP

Method	OCA		OCN	
	**Mean value (mm**^**2**^**)**	**ICC(95% confidence interval)**	**Mean value**	**ICC(95% confidence interval)**
SA	58.604	0.909 (0.784 to 0.962)	38.477	0.929 (0.832 to 0.970)
DP	48.759	0.906 (0.767 to 0.961)	37.750	0.904 (0.772 to 0.960)

Abbreviations. *OCA* Occlusal contact areas, *OCN* Occlusal contact numbers, *SA* Scanning of articulating paper marks, *DP* Dental prescale occlusion analysis system

### Evaluation of the validity of SA and DP

**Fig. 5:**
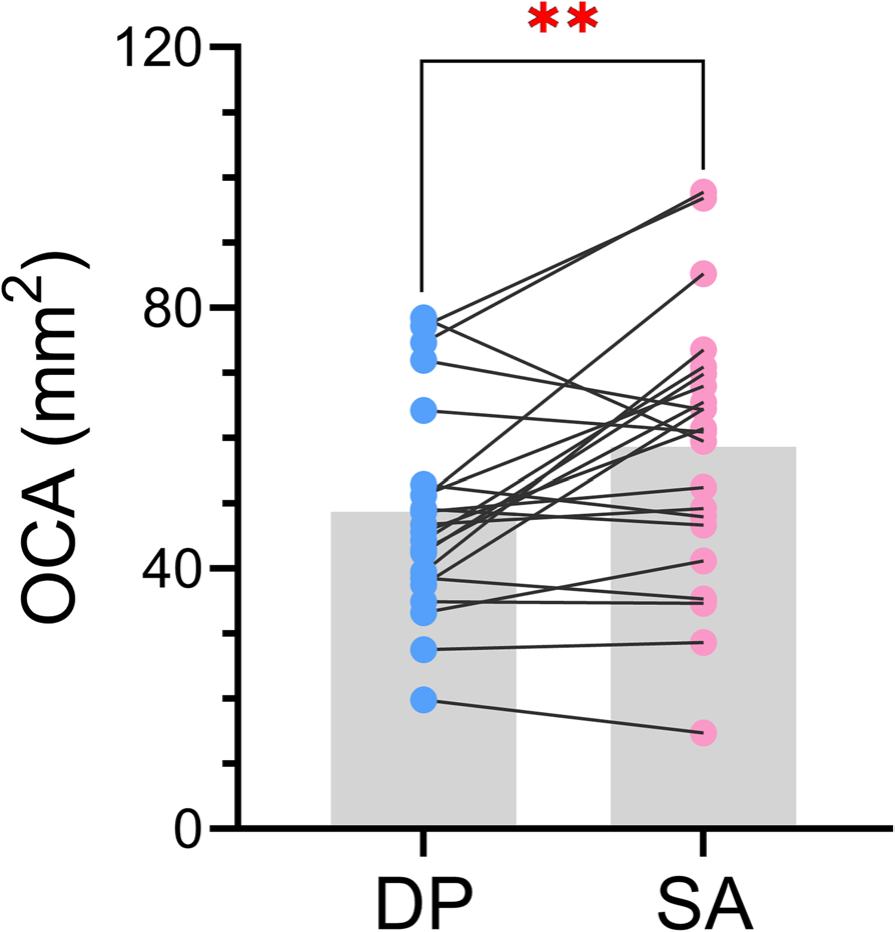
Results of the paired t-test between the OCA obtained from DP and SA

**Fig. 6:**
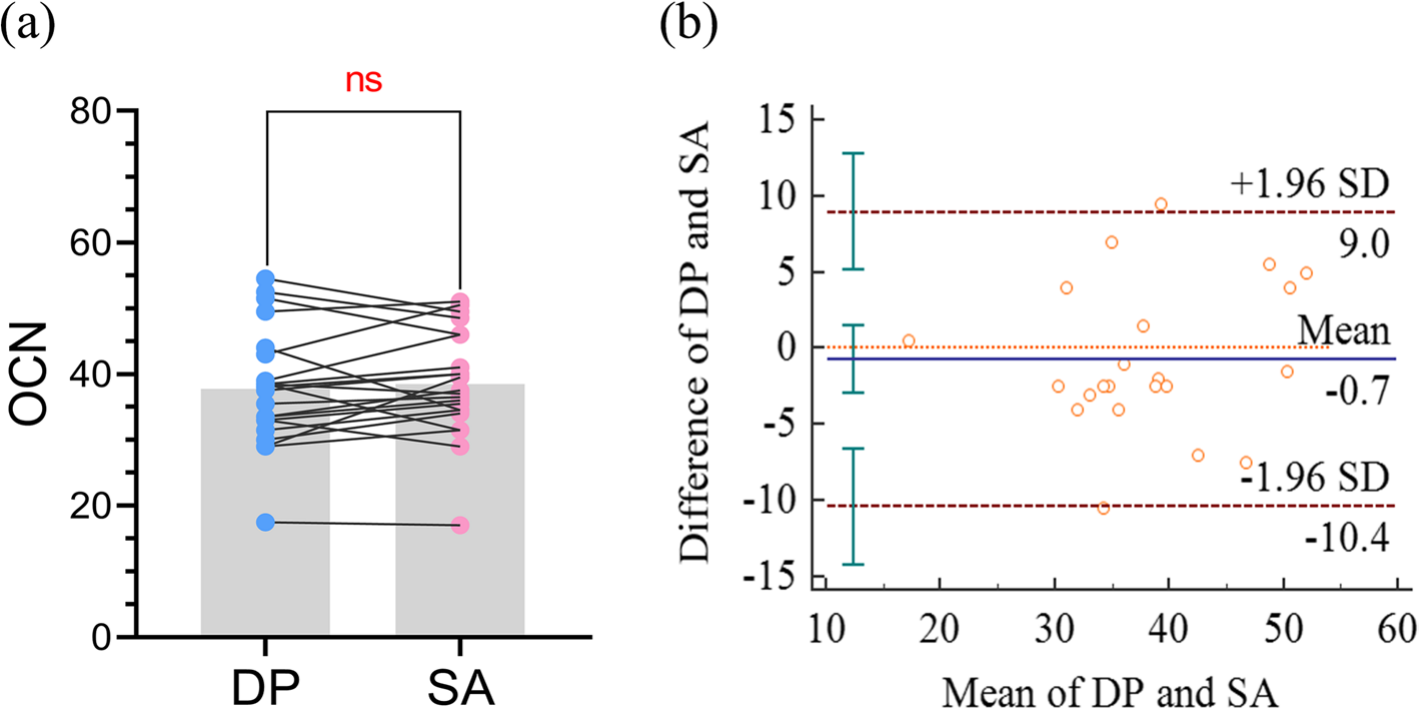
Paired t-test and Bland–Altman plot for the OCN. **a**. Results of the paired t-test between the OCN obtained from DP and SA. **b**. Bland–Altman plot illustrating the agreement between the OCN obtained from DP and SA

The results of the paired t-test showed a significant difference between the OCA obtained from the two systems (t = 3.017, *P* < 0.01) (Fig. 5), while there was no significant difference between the OCN obtained from the two methods (t = 0.691, *P* > 0.05) (Fig. 6a).

For data with no statistically significant differences in the paired t-tests, the Bland–Altman analysis showed that all data except one fell within the 95% limit of agreement of the two methods, which indicated that DP is consistent with SA in measuring OCN (Fig. 6b). Pearson correlation analysis showed a significant correlation between the OCA obtained from DP and SA analysis (*R* = 0.68, *p* < 0.001).

Based on these results, it can be seen that there is good consistency between DP and SA methods, therefore it is feasible to combine them to construct a 3D occlusal force analysis system.

### Occlusal forces by 3D occlusal force analysis

**Fig. 7:**
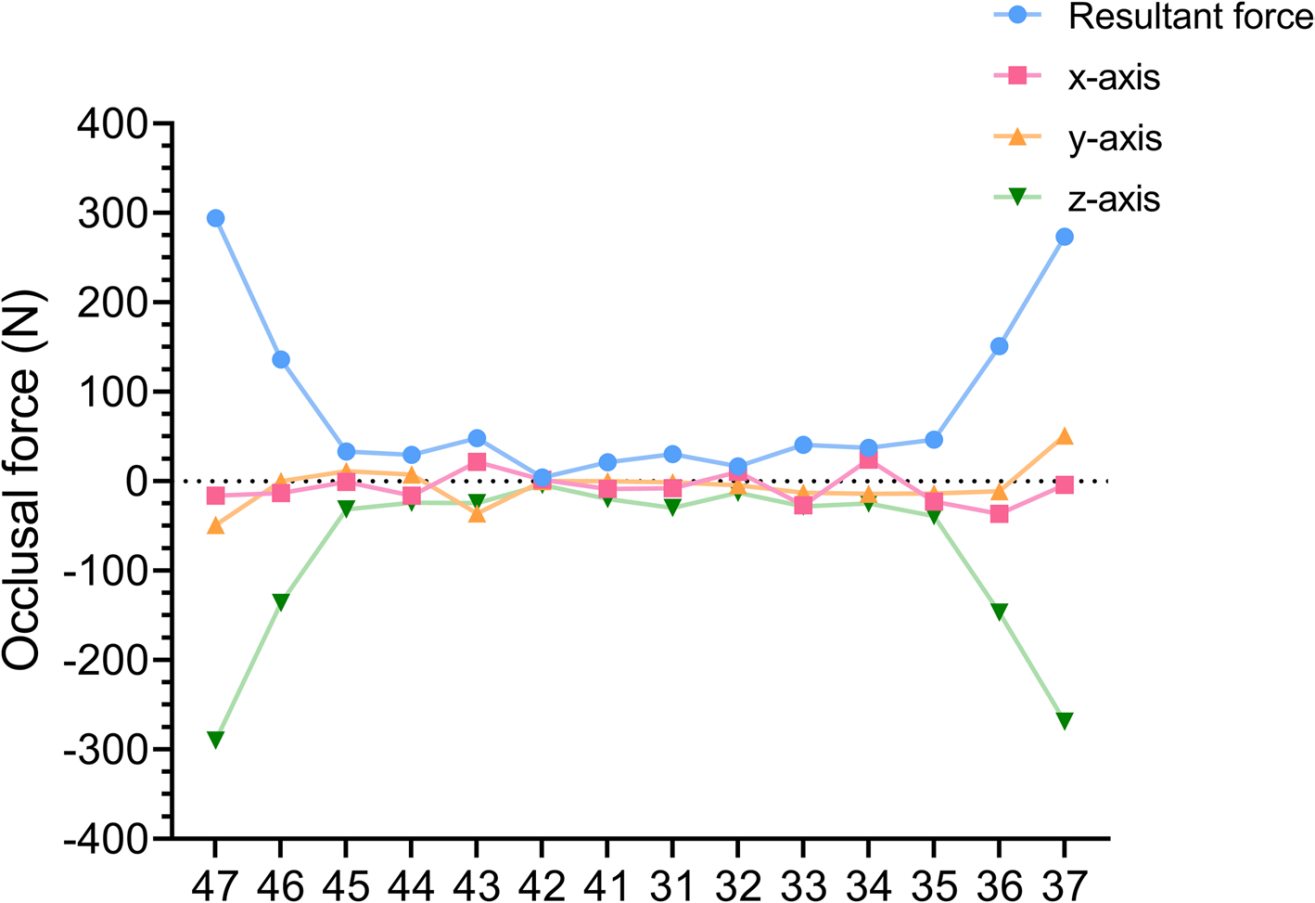
The resultant force and the component forces on the x-, y- and z-axes of the occlusal force in each tooth position

Analysis of the occlusal forces in this patient showed that the occlusal forces changed in response to tooth position. The occlusal force in the anterior region < premolar region < molar region, both in terms of the resultant force and in terms of the *x*-, *y*- or *z*-axis components (Fig. 7). In addition, the forces on the individual teeth are clearly illustrated above. The vertical forces point in the negative direction of the z-axis, while the *x*- and *y*-axis components point in different directions.

## Discussion

Occlusal contact analysis, including the location of contact, OCA, OCN and occlusal force of each tooth, is essential in dentistry, especially in prosthetics and orthodontics, as maintaining the balance of occlusal contact and force of the teeth or prosthesis is the goal of the occlusal adjustment. However, there is still a lack of a convenient and reliable method which can achieve the abovementioned occlusal contact information simultaneously. In this study, after verifying the consistency between the 2D and 3D occlusion analysis systems, we have developed a novel occlusion analysis method to locate occlusal contact and quantitatively analyze OCA, OCN and occlusal forces of each tooth.

In evaluation trial, the reliability and validity of two analysis techniques were evaluated. The results indicated that the ICC values obtained from two systems were higher than 0.9, which indicated high stability for both two systems. Paired t-tests demonstrated no significant difference in the OCN values obtained from the DP and SA analysis. The following Bland–Altman test showed good consistency between the results of the two systems. Therefore, it can be considered that these two methods have a high consistency in measuring the OCN. In measuring OCA, although paired t-test showed that a significant difference between the data obtained from these two analysis systems, a significant correlation was indicated by Pearson correlation analysis. The result that the OCNs were the same while the OCAs were proportionally enlarged may be due to the influence of the media used in the analysis systems. The pressure sensitive film of DP comprises three layers of PET, which is more resistant to compression deformation than articulating papers. In addition, the dye of the articulating paper has spreading ability in the presence of saliva [30, 31]. Thus, the stained area of articulating paper might extend beyond the actual occlusal contact area. From this point of view, the difference OCA values did not affect the distribution of the occlusal contact as similar OCN values were found. In clinical demonstration, analysis of the occlusal forces showed that the occlusal forces changed in response to the tooth position. Usually, the first molar is subjected to the greatest force. However, for this patient with bruxism, the second molar is subjected to the greatest occlusal force during occlusion. This anomaly results in more wear of the cusps of the second molar. Furthermore, we can clearly see from the data how the teeth are subjected to forces in different directions. For example, the patient's second molar teeth on the left and right side have opposite directions of force in the *y*-axis, which cannot be identified in a two-dimensional force analysis. Many oral diseases are caused by imbalance in occlusal forces and it is easy to misdiagnose the two forces as the same based on the force values alone. The identification of the direction of force will be of great help in the treatment and diagnosis of diseases.

The difference between the 2D analysis results and the 3D dentition, caused by the loss of spatial information, will cause inconvenience in the clinical application of these occlusal analysis systems [32]. For example, quantitative analysis results (OCA or occlusal contact force) from T-scan and DP can not be used directly to locate the occlusal contact on the surface of teeth in clinical practice. In recent years, with the introduction of digital technology into dental practice, some 3D occlusal analysis systems were designed [33, 34]. For example, the occlusal contact analysis function that comes with the intraoral scanner, the transillumination technique used in the study by DeLong et al. [32] and the virtual occlusion analysis system proposed by Li et al. [35]. These methods achieved quantitative analysis of the OCA in three dimensions, however, they did not include the function of occlusal force analysis. After verifying the consistency between the two systems in detect occlusal contact, we constructed a 3D occlusal analysis method system by combining the advantages of respective systems. This new method not only achieved a 3D spatial location of occlusal contact points, but also combined the force analysis function to make a powerful and quantitative evaluation of occlusal contact of nature teeth or prosthesis. These innovations provide clinicians with more realistic and comprehensive information on occlusal contact, which would improve the accuracy of clinical diagnosis and treatment, as well as improve their clinical decision-making ability.

Another potential advantage of this novel occlusal analysis method is that it is more accurate and flexible in occlusal force analysis. It can not only decompose the bite force, but also specify the direction of force division as required. These characteristics have not been reported in previous studies. Prado et al. [36] applied T-scan to three-dimensional dentition analysis with the help of oral appliances. However, oral appliances increase the inconvenience of occlusion collection. In addition, due to the limitations of the T-scan [37], it can only display the relative value of the force and cannot obtain the absolute magnitude of the occlusal force, let alone the decomposition of the occlusal force. The study by Hattori et al. proposed a method to set the occlusal force value and direction and initially constructed a 3D occlusal force analysis system. However, its arithmetic is quite complicated and cannot be flexibly adjusted to clinical needs [38]. In our study, the constructed method could allow analytical calculations of the magnitude of the occlusal forces in the proximal-distal or buccolingual directions or any other direction required, as well as the magnitude of the fractional forces on individual tooth or combinations of several teeth in different directions, as required. It is worth noting that the calculation of the magnitude of forces in directions specific direction, such as proximal, distal, buccolingual and others, is of great importance in clinical practice, especially in prosthetic treatment, because abnormal forces can lead to occlusal instability and damage.

In addition, as the pressure-sensitive film and intraoral scanning techniques used in this method are simple to operate, this new occlusal analysis method has the potential advantages of simple operation and intuitive analysis results. Occlusal contact analysis methods in previous studies are unable to visualize the direction and magnitude of the occlusal forces. In the constructed method in this study, the length of the line segment to simulate the magnitude of the occlusal force and the direction of the line segment to illustrate the direction of the occlusal force. This allows for a more visual presentation of the results of the occlusal analysis and facilitates clinical work, for example, in presenting and explaining the abnormal occlusal conditions to patients.

Although the new method system has several potential advantages mentioned above, there are also some limitations that need to be further studied. At this stage, this 3D occlusal force analysis method needs to be constructed manually and the simulation of occlusal forces at each occlusal contact point repeats the same operational steps. In future research, we will try to apply artificial intelligence and other methods to realize the automation of these operations, and further improve the efficiency and automation level of the new method. Besides, the inability to obtain dynamic occlusal force changes throughout mandibular movements is also the limitation of the system. To address this issue, more computer-aided simulation and occlusal analysis tools are needed.

## Conclusion

In summary, this study constructed a novel occlusion analysis method which can not only locate the occlusal contact in teeth or prosthesis, but also quantitatively analyze the OCA, OCN and occlusal forces simultaneously. The magnitude and direction of occlusal force of each contact point can be intuitively displayed on the 3D model with line segments. This new method would help to promote the accuracy of clinical diagnosis and treatment, especially in the field of prosthodontics, implants, and orthodontics.

## Data Availability

Data that support the findings of this study are available from the corresponding author [F.W.], upon reasonable request.

## Abbreviations

SA: Scanning of articulating paper marks
DP: Dental Prescale analysis system
ICC: Intraclass correlation coefficient
ICP: Intercuspal position
OCA: Occlusal contact area
OCN: Occlusal contact number
PET: Polyethylene terephthalate
